# Characterization and Evolutionary Analysis of a Novel H3N2 Influenza A Virus Glycosylation Motif in Southern China

**DOI:** 10.3389/fmicb.2020.01318

**Published:** 2020-06-16

**Authors:** Zhiqi Zeng, Lee-Fong Yau, Zengxian Lin, Xuanzi Xia, Zifeng Yang, Jing-Rong Wang, Wenjun Song, Xinhua Wang

**Affiliations:** ^1^State Key Laboratory of Respiratory Disease, National Clinical Research Center for Respiratory Disease, Guangzhou Institute of Respiratory Health, The First Affiliated Hospital of Guangzhou Medical University, Guangzhou, China; ^2^State Key Laboratory of Quality Research in Chinese Medicine, Macau Institute for Applied Research in Medicine and Health, Macau University of Science and Technology, Macau, China; ^3^Institute of Integration of Traditional and Western Medicine, Guangzhou Medical University, Guangzhou, China

**Keywords:** A(H3N2), receptor binding, antibody recognition, glycosylation possessing, glycoforms

## Abstract

An influenza A (H3N2) virus epidemic occurred in China in 2017 and the causative strain failed to bind red blood cells (RBCs), which may affect receptor binding and antibody recognition. The objective of this study was to analyze the genetic characteristics and glycosylation changes of this novel H3N2 strain. We directly sequenced the hemagglutinin (HA) genes of H3N2 clinical specimens collected from patients with acute respiratory tract infection during 2017 in Guangdong, China. We aligned these sequences with those of A/Hong Kong/1/1968 (H3N2) and A/Brisbane/10/2007 (H3N2). Glycosylation changes were analyzed by C18 Chip-Q-TOF-MS. A/China/LZP/2017 (H3N2) was negative by HA assay, but was positive by quantitative real-time Polymerase Chain Reaction (qPCR) and direct immunofluorescence assay (DFA). We found that the HA1 residue 160T of A/China/LZP/2017 (H3N2) could block virus binding to receptors on RBCs. Furthermore, the ASN (N)-X-Thr (T) motif at HA1 residues 158–160, encoding a glycosylation site as shown by C18 Chip-Q-TOF-MS, predominated worldwide and played a critical role in RBC receptor binding. Ten glycoforms at HA1 residue 158 were identified [4_3_1_0, 5_6_0_1, 3_3_0_1, 4_4_3_0, 6_7_0_0 (SO_3_), 3_6_2_0, 4_3_1_2 (SO_3_), 7_5_2_0 (SO_3_), 3_6_2_1 (SO_3_), and 3_7_0_2]. Glycosylation changes at HA1 residues 158–160 of a circulating influenza A (H3N2) virus in Guangdong, China, in 2017 blocked binding to RBC receptors. Changes to these HA1 residues may have reduced protective antibody responses as well. Understanding these critical epitopes is important for selecting vaccine strains.

## Introduction

Influenza A virus H3N2 first emerged in 1968, and frequently leads to annual outbreaks within the human population (Nair et al., 2011). Important contributions to the pandemic of influenza have come from hemagglutinin (HA), which is a major surface antigen of the virus. Mutations in the HA may cause affecting receptor binding and immune response. Thus, understanding the novel mutation is critical for developing strategies to control the pandemic of influenza A (H3N2).

The hemagglutinin (HA) protein plays an important role in binding host cell receptors and releasing the viral RNA into the cell (Beer et al., 2018). HA is composed of two polypeptides, HA1 and HA2. HA1 contains the receptor binding site in a globular head domain and targets sialic acid residues on host cells. HA2 forms a stem-like structure containing a fusion domain (N-terminal) and a transmembrane anchor domain (C-terminal), and mediates fusion of the virus envelope and endosomal membrane (Skehel and Wiley, 2000). Both regions contain N-linked oligosaccharides (Keil et al., 1985), which may interfere with the function of HA. The majority of oligosaccharides are conserved and promote fusion activity. But glycans near the antigenic epitopes may influence the antibody recognition and those near the proteolytic activation site may affect the infectivity of the influenza virus (Skehel et al., 1984; Deshpande et al., 1987).

HA mutations of influenza A (H3N2) have been emerging with changes of glycosylation since 1968. Influenza A (H3N2) has re-assorted with an H2N2 virus, the former virus is capable of transmission between humans (Westgeest et al., 2014; Poucke et al., 2015). The H3N2 virus mutated so quickly that the World Health Organization (WHO) has recommended 28 vaccine strains (Lin et al., 2017). The rapid mutation rate of the H3N2 virus has been proposed to lead to generation of a novel virus in 2–5 years (Park et al., 2009). In the 2014/15 season, two new clades started to emerge. Clade 3c.2a, characterized by the F159Y and K160T mutations, bore a novel potential glycosylation site (Chambers et al., 2015; Skowronski et al., 2016). Another clade in circulation was 3c.3a (including strain A/Switzerland/9715293/2013) (Chambers et al., 2015). During the 2016/17 season, clade 3c.2a separated into two new clades. The 3c.2a1, characterized by amino acid changes such as T135K, N171K, and D122N, may have resulted in the loss of a glycosylation site (Melidou et al., 2017). By the 2017/18 season, clade 3c.2a2 began to split into cluster I, bearing the I58V and S219K mutations, and cluster II, bearing the N122D and S262N mutations. The N122D mutation resulted in the loss of a glycosylation site (Harvala et al., 2017). Generally, the HA1 genes of H3N2 viruses undergo continuous mutations result in losing or possessing of glycosylation to reduce host immune recognition and to enhance transmissibility (Hensley et al., 2009; Popova et al., 2012). The majority of neutralizing antibodies induced by vaccination or infection do not target the receptor-binding site (RBS), which is surrounded by highly variable regions of HA1, and prevent virus binding to cell receptors (Gerhard et al., 1981; Knossow et al., 1984; Whittle et al., 2011).

Besides, the HA protein is critical for the virulence and host preference as it binds to sialic acid receptors on epithelial cells (Shinya et al., 2006; de Wit et al., 2010). It was reported that mutation of HA glycosylation sites can affect the specificity and affinity for receptor binding (Mair et al., 2014). As the amount of glycans becomes more, the binding ability for the receptor is weakened (Abe et al., 2004). However, influenza will undergo multiple mutations to make a successful adaptation to humans.

Overall, the number and position of *N*-linked glycans in HA have influenced the evolution of H3N2 viruses and can contribute to immune evasion (Wanzeck et al., 2011; Tate et al., 2014). In our study, we identified H3N2 clinical strains from Guangdong, China, in 2017 that were not able to bind RBCs. The surface of RBCs contains many receptors for binding by HA. RBCs clump together because of interactions between the HA of virus particles and RBCs, leading to a lattice formation, which called the hemagglutination assay (Grimes, 2002). This suggested that H3N2 virus epitopes may change and influence binding to host cell receptors as well as antibody responses. Therefore, it is necessary to provide further insights into the genetic characteristics and glycosylation changes of H3N2 viruses.

## Materials and Methods

### Cells and Viruses

Madin-Darby canine kidney (MDCK) cells were purchased from the American Tissue Culture Collection (ATCC). The cells were cultured in minimum Eagle’s medium containing 10% heat-inactivated fetal bovine serum. Influenza A viruses A/Brisbane/10/2007 (H3N2) (B10) and A/Hong Kong/4801/2014 (H3N2) (HK4801) were also purchased from the ATCC. Influenza A viruses A/China/LZP/2017 (H3N2) LZP) and A/China/GMU-SKLRD03/2017 (H3N2) (GMU03) were detected and isolated from clinical specimens. Collection of clinical specimens was approved by the Ethics Committee of The First Affiliated Hospital of Guangzhou Medical University (2014 No. 50; Guangzhou, China; File number: 2014-50, date: June 2, 2015). Written informed consent was obtained from study participants.

### Viral Genome Characterization and Reverse Genetics

RNA of the four H3N2 strains was extracted and reverse transcribed. The sequences of LZP and GMU03 were deposited in GenBank (LZP: Accession numbers MK053902 to MK053909; GMU03: Accession numbers MN658587). The eight viral segments of different strains were amplified and cloned into a pHW2000 plasmid system as previously described (Wang et al., 2019). The seven segments of LZP (NA, PB1, PB2, PA, NP, M, NS) were used as a backbone to rescue recombinant viruses (LZP-B10, LZP-HK4801, LZP-GMU03) bearing the HA genes of B10, HK4801, and GMU03, respectively.

### Hemagglutination Assay

The hemagglutination assay was performed in a V-bottom 96-well microtiter plate. The most concentrated sample was in the first well, and subsequent wells were serially diluted twofold in phosphate-buffered saline (PBS). The first row served as a negative control and contained only PBS. Different strains of virus were detected by 0.5% solution of turkey RBCs.

### Quantitative Real-Time PCR (qPCR)

RNA from wild type and recombinant H3N2 viruses was extracted using the QIAamp Viral RNA Mini Kit (Qiagen, Germany) and analyzed by qPCR using the ABI 7500 Real-time PCR System (Wang et al., 2019).

### Direct Immunofluorescence Assay (DFA)

The nucleoproteins (NPs) of H3N2 viruses were detected using the D3 Ultra DFA Respiratory Virus Screen and ID Kit (Healthline Media UK Ltd., Brighton, United Kingdom). H3N2 strains (LZP, GMU03) and B10- or HK4801-infected cells were fixed in 4% paraformaldehyde at 37°C for 30 min. Fluorescein isothiocyanate (FITC)-labeled mouse anti-NP antibody was used to detect nucleoprotein. The cells were observed using a fluorescence microscope (Zeiss Axiovert 135, Zeiss, Oberkochen, Germany).

### Bioinformatic Analysis

The HA genes of B10 and HK4801 were obtained from the National Center for Biotechnology Information (NCBI) Influenza Virus Resource Database and the HA genes of LZP and GMU03 were sequenced by Sangon Biotech (Shanghai, China). Both sequences were analyzed using Vector NTI (Invitrogen, United States). The prevalence of the T160K, N158K and the substitutions at residues 158–160 were analyzed through the Global Initiative on Sharing All Influenza Data (GISAID) database^1^.

### Mutation of H3N2 HA1 N158K and T160K

Mutations were introduced at HA1 residues 158 and 160 using the QuikChange site-directed mutagenesis kit (Agilent Technologies, Santa Clara, United States). HA1-N158K and HA1-T160K mutant viruses were rescued as described previously (Hoffmann et al., 2000).

### Enzymatic Digestion of Influenza Proteins

Influenza proteins were purified by filtration through a 10-kDa cutoff centrifugal filter, and sequentially treated with 10 mM dithiothreitol and 2 M hydroxylamine. The samples were alkylated with 55 mM iodoacetamide. Next, the samples were concentrated and buffer-exchanged buffer into H_2_O using a 3-kDa cutoff centrifugal filter. The protein concentration was determined using a Bio-Rad protein assay.

Fifty micrograms of protein were diluted with 100 mM Tris-HCl containing 10 mM CaCl_2_ (pH 8.0) to a final concentration of 1 μg/μL. Two micrograms of chymotrypsin were added and incubated for 16 h incubation at 25°C. The digested samples were loaded onto the preconditioned porous graphitic carbon cartridge and targeted glycopeptides were eluted with 50% acetonitrile (ACN) containing 0.1% trifluoroacetic acid. The collected fraction was dried using a speed vacuum. Finally, the dried residue was reconstituted in 50 μL of MilliQ H_2_O prior to analysis.

### Glycopeptide Profiling by C18 Chip-Q-TOF-MS

Chromatographic separation of glycopeptides was performed on an Agilent 1260 Infinity HPLC Chip LC system (Agilent, Santa Clara, CA, United States) equipped with a Polaris-HR C18 chip (G4240-62030, Agilent). Three microliters of each sample were loaded onto the enrichment column in a solution of 0.1% formic acid (FA) and 3% ACN in water at 3 μL/min. The mobile phase used in the nano-pump consisted of 0.1% FA in water (A) and 0.1% FA in ACN (B). The gradient was as follows: 3–40% B for 15 min, 40–70% B for 1 min, 70% B for 1 min, 70–3% B for 0.5 min, and 3% B for 10 min. The flow rate was 0.45 μL/min.

Glycopeptide profiling was carried out on an Agilent 6550 iFunnel Q-TOF MS. The dry gas (N_2_) was flowed at 11 L/min at 250°C. MS spectra were acquired in positive mode with the mass range m/z 450–3000. Mass correction was enabled using reference masses of m/z 922.0098.

### Ethics Approval and Consent to Participate

All clinical specimens have been approved from the Ethics Committee of The First Affiliated Hospital of Guangzhou Medical University (2014 no. 50; Guangzhou, China) (File number: 2014-50 date: June 2, 2015). Written informed consent was obtained from the participant.

## Results

### Detection of H3N2 Clinical Specimens

**FIGURE 1:**
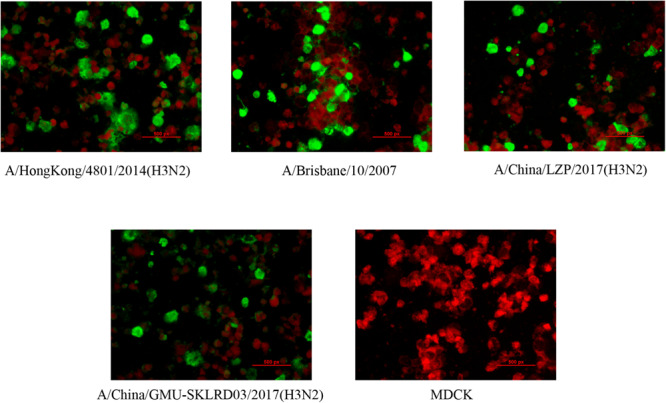
Immunofluorescence assay for detecting H3N2 clinical specimens. The nucleoproteins (NPs) of H3N2 viruses were determined by the D3 Ultra DFA Respiratory Virus Screen and ID Kit. H3N2 strains (LZP, GMU03) and B10- or HK4801-infected cells were fixed in 4% paraformaldehyde at 37°C for 30 min. FITC-labeled mouse anti-NP antibody was used to detect nucleoprotein. The cells were observed using a fluorescence microscope.

LZP and GMU03 were negative by hemagglutination assay. The positive controls, B10 and HK4801 were positive and their hemagglutination titers were 1:64 and 1:512, respectively (Table 1 and Supplementary Figure S1). To determine whether the two clinical isolates were truly negative for hemagglutination activity, we used qPCR and DFA. The CT values of LZP and GMU03 were 21.85 and 23.14, respectively, by qPCR (Table 1). The viral NPs of the H3N2 viruses were detected by DFA using a FITC-labeled mouse anti-NP antibody (Figure 1).

**TABLE 1:** qPCR for detecting HA in H3N2 clinical specimens.

Species of virus	Hemagglutination assay	qPCR (CT values)
A/China/LZP/2017 (H3N2)	(–)	21.85
A/China/GMU-SKLRD03/2017 (H3N2)	(–)	23.14
A/Brisbane/10/2007	(+) 1:64	15.08
A/Hong Kong/4801/2014 (H3N2)	(+) 1:512	14.24

*(+), positive; (–), negative. Throat swabs (LZP, GMU03) were collected from patients with acute respiratory tract infection (ARTI) in Guangdong, China during 2017. The viral RNA/DNA was extracted and reverse transcribed. The HA of A (H3N2) was determined by qPCR kit. Meanwhile, the clinical specimens were tested using the HA assay.*

### Determination of Critical Viral Proteins

**FIGURE 2:**
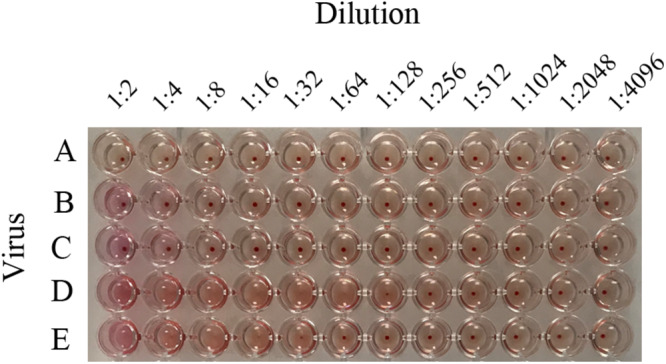
Hemagglutination titers of H3N2 recombinant viruses. To figure out whether HA protein of LZP led to unable to bind the RBCs, the seven segments of LZP (NA, PB1, PB2, PA, NP, M, NS) were used as a backbone to rescue recombinant viruses (LZP-B10, LZP-HK4801, LZP-GMU03) bearing the HA genes of B10, HK4801, and GMU03, respectively. The recombinant viruses and LZP were determined by HA assay. A: PBS; B: LZP; C: LZP-GMU03; D: LZP-B10; E: LZP-HK4801.

To confirm whether the HA protein affected the binding ability of viruses to RBCs, LZP-03, LZP-B10, and LZP-4801 H3N2 viruses were rescued as recombinant viruses. LZP and LZP-GMU03 recombinant viruses were both negative by hemagglutination assay (Figure 2). However, the LZP-B10 and LZP-HK4801 recombinant viruses showed hemagglutination activity at titers of 1:64 and 1:16, respectively. Thus, negative results in hemagglutination assays were caused by the HA proteins of H3N2 viruses.

### Identification of Mutated Sites in A/China/LZP/2017 (H3N2)

To identify important mutations, the HA sequences of LZP and GMU03 were compared with those of B10 and HK4801. Two mutations in the HA protein were found: HA1-K121N and HA1-T160K.

### Critical Nature of the N158K and T160K Mutations in A/China/LZP/2017 (H3N2)

**FIGURE 3:**
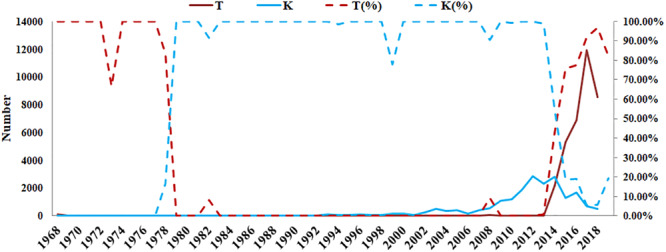
Prevalence of T and K residues at HA1 position 160 in the world since 1968. The sequences of 62330 clinical strains worldwide from 1968 to 2019 were collected from GISAID. The T and K residues at HA1 position 160 were analyzed. The y-axis on the left: the number of HA1-160T and HA1-160K; The y-axis on the right: the percentage of HA1-160T and HA1-160K.

**FIGURE 4:**
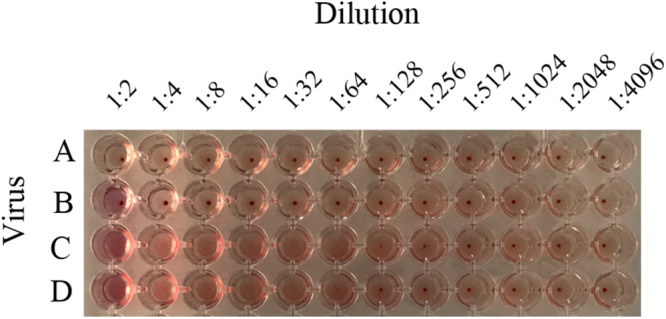
Hemagglutination titers of H3N2 mutant strains. To distinguish whether the possessing of glycosylation motif site at position 158 failed to bind RBCs, recombinant virus containing HA1-160K, HA1-158K, and LZP were determined by HA assay. A: PBS; B: LZP; C: T160K; D: N158K.

**FIGURE 5:**
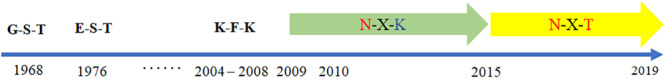
Worldwide prevalence of the glycosylation motif site at position 158 from 1968 to 2019. The sequences of 62,330 clinical strains worldwide from 1968 to 2019 were collected from GISAID. The substitutes at position 158–160 were analyzed.

**FIGURE 6:**
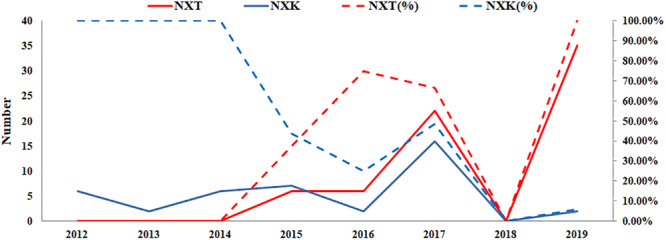
Prevalence of the N-X-T/K motif in Guangdong, China from 2012 to 2019. The sequences of 106 clinical strains in Guangdong, China from 2012 to 2019 were collected from GISAID. The substitutes N-X-T/K at position 158–160 were analyzed. The y-axis on the left: the number of the substitutes N-X-T and N-X-K at HA1 residues 158–160; The y-axis on the right: the percentage of the substitutes N-X-T and N-X-K at HA1 residues 158–160.

According to the GISAID database, a Thr substitution at HA1 residue 160 H3N2 viruses rose in frequency from 2013 to the present day. By contrast, the peak frequency of K160 occurred in 2012 (2848/2850 isolates, 99.93%) and then gradually declined (Figure 3). After incorporating a T160K mutation in LZP, the hemagglutination assay was positive with a titer of 1:64. However, the frequency of K160 started to rise in 2018. In addition, another critical substitution of LZP was identified at HA1 residue 158. When Asn was substituted with Lys at this position, a positive result was also obtained in the hemagglutination assay (Figure 4). Both N158K and T160K of LZP could change the binding ability to RBCs. From 2013 to present, the frequency of the N-X-T motif at residues 158–160 increased worldwide and became dominant in 2015 (5198/6805 isolates, 76.4%), where X is any amino acid except Pro. In Guangdong, China, the N-X-T motif began to dominate in 2016 (6/8 isolates, 75%) (Figures 5, 6).

### Glycopeptide Profiling of HA1 Residue 158

**FIGURE 7:**
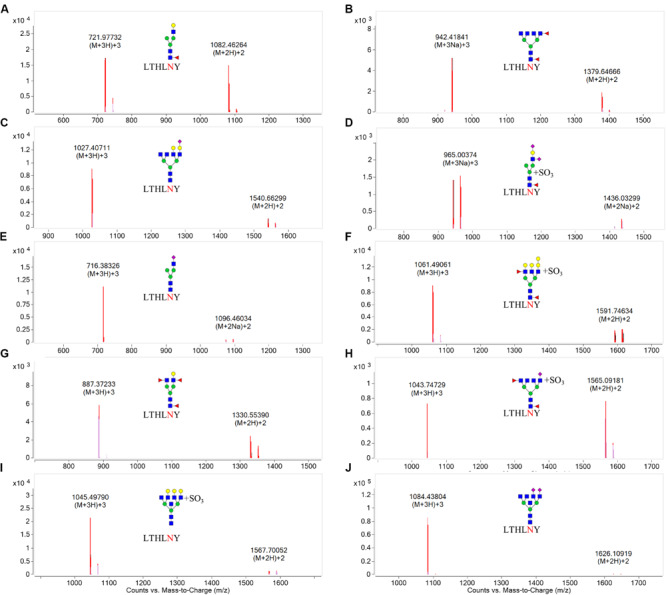
Overlaid extracted compound chromatograms (ECCs) of identified HA1 glycopeptides containing the glycosylation site at position 158. HA1 proteins extracted from influenza were digested with chymotrypsin at 25°C for 16 h, followed by profiling the glycopeptides by C18 chip-Q-TOF-MS. The glycoforms at position 158 were then identified based on high-resolution MS data. **(A)**: 4_3_1_0, **(B)**: 3_6_2_0, **(C)**: 5_6_0_1, **(D)**: 4_3_1_2 (SO3), **(E)**: 3_3_0_1, **(F)**: 7_5_2_0 (SO3), **(G)**: 4_4_3_0, **(H)**: 3_6_2_1 (SO3), **(I)**: 6_7_0_0 (SO3), **(J)**: 3_7_0_2.

N-linked glycans can only be attached to the nitrogen atom of an N residue that forms part of the consensus sequence N-X-Ser(S)/T. Therefore, one chymotryptic glycopeptide at residues 154–159 (peptide sequence: LTHLNY) of HA, which possessed a glycosylation site at HA1 residue 158, was identified in LZP. Based on the high-resolution MS data and matching with the database, 10 glycoforms were identified at residue HA1 position 158 including three neutral glycans (3_6_2_0, 4_3_1_0, and 4_4_3_0), three acidic glycans (5_6_0_1, 3_3_0_1, and 3_7_0_2), and four sulfated glycans [6_7_0_0 (SO_3_), 4_3_1_2 (SO_3_), 7_5_2_0 (SO_3_), and 3_6_2_1 (SO_3_)] (Figure 7). Both the N158K and T160K mutations resulted in loss of this glycosylation site.

## Discussion

Influenza is a global threat and represents a pressing public health challenge worldwide (Schoenbaum, 2004). In China, an outbreak of influenza A H3N2 occurred during 2017 (Supplementary Figure S2). We found that some H3N2 strains in Guangdong did not bind RBCs and tested negative by hemagglutination assay. To confirm this result, we collected several clinical H3N2 strains, along with B10 and HK4801 as positive controls, and analyzed their genetic characteristics. As HA protein is present on enveloped viruses such as influenza viruses, it is able to bind receptors on the membranes of RBCs to cause agglutination (Grimes, 2002). By inserting the HA genes of GMU03, B10, HK4801 into an LZP backbone, we were able to use a hemagglutination assay to deeply analyze the HA proteins of LZP and GMU03. We found that HA1 121K and 160T may play an important role in binding to RBCs. According to the GISAID database, the frequency of HA1 K160 has risen since 2017 worldwide, but T160 was still dominant in these years. Another residue, HA1 158, was also investigated. We mutated N158K and T160K and found that both strains were positive by HA assay, which suggested that mutations at both residues were responsible for failure to bind RBCs. In Guangdong Province, more isolates bore N-X-T substitutions at HA1 residues 158–160 during 2017. Further, we found that HA1 residue 158 of LZP bore an *N*-linked glycosylation site by enzymatic digestion of influenza proteins and C18 Chip-Q-TOF-MS analysis. In summary, our study confirmed that glycosylation at HA1 residues 158–160 can block receptor binding to RBCs.

It was reported that the loss and possessing of the glycosylation sites in the head region could affect recognition by antibodies. During the 2016/2017 season, Melidou et al. (2017) demonstrated that the T135K, N171K, and D122N substitutions were located within antigenic sites and potentially affected viral antigenicity, as they resulted in losses of *N*-linked glycosylation sites. Furthermore, the I58V and S219K mutations of clade 3c.2a2, cluster I and the N122D and S262N mutations of cluster II were found to result in the loss of a glycosylation site during the 2017/18 season (Harvala et al., 2017). Nevertheless, since transmitting between the human population, HK/68 have gained up to seven glycosylation sites on the HA globular head and five on the stem from the only original two sites (Yang et al., 2015; Alymova et al., 2016). In our study, we found that strains encoding 160T and 158N began to dominate in Guangdong Province, China during the 2015/16 season, leading to a new potential glycosylation site. Similar to previous studies, they concluded that the glycosylation site was a crucial factor affecting the antibody response. Cueno et al. (2019) demonstrated that residues 158–160 in the H3N2 hemagglutinin globular head had structural significance for antigenic drift by modeling. Moreover, Beer et al. (2018) screened the epitopes of anti-A/Victoria/361/2011 neutralizing mouse monoclonal antibodies (mAbs) and found that HA1 residue 158 was glycosylated leading to reduce binding by mAbs. Zost et al. (2017) also found that the generation of protective antibody responses against a H3N2 vaccine strain was affected during the 2016–2017 H3N2 influenza season due to the substitutions within HA1 residue 160 resulting in a new glycosylation site in the HA protein, which affected antigenicity. Generally, the addition or deletion of glycosylation sites on the globular head is vital for escaping the immune response of human. We supposed that the possessing of the glycan at HA1 residue 158 may be a important contribution for antigenic drift in Guangdong, China in recent years.

Not only the glycosylation site, the length and the type of the glycans on the HA head region may be other reasons underlying our observations. In our study, we discovered 10 different glycoforms at residue 158 [4_3_1_0, 5_6_0_1, 3_3_0_1, 4_4_3_0, 6_7_0_0 (SO_3_), 3_6_2_0, 4_3_1_2 (SO_3_), 7_5_2_0 (SO_3_), 3_6_2_1 (SO_3_), and 3_7_0_2]. As the length of the glycans, Cheng-Chi et al. (2009) reported that antibodies raised against the fully glycosylated HA were less binding affinity to antibody than bearing a single N-linked GlcNAc at each glycosylation site and implicated that removal of structural and non-essential glycans on viral glycoproteins might be an effective approach for vaccine design. Melissa Boeijen reviewed that long oligosaccharide side chains can block the binding of neutralizing antibody due to coverage of the antibody binding site and mentioned that galactose and fucose were long glycans (Boeijen, 2013). In the study, the length of glyforms were ranked as followed: 7_5_2_0 (SO_3_) > 4_3_1_2 (SO_3_), 5_6_0_1 > 4_3_1_0, 4_4_3_0, 6_7_0_0 (SO_3_) > 3_3_0_1, 3_6_2_1 (SO_3_), 3_7_0_2 > 3_6_2_0. Therefore, we suggested that 7_5_2_0 (SO_3_) should be highly concerned. Additionally, the type of oligosaccharides side chains is critical as well to result in antigenic drift. A recent study by An et al. (2019) supposed that the globular head region with high-mannose and complex glycans would promote the virus well shielding under the immune system. As we found, 3_6_2_0, 5_6_0_1, 7_5_2_0 (SO_3_), 3_6_2_1 (SO_3_), 6_7_0_0 (SO_3_), 3_7_0_2 are large, complex glycans on the N158 glycosite. Thus, not only the length but also the types of glycoform are crucial factors to affect immune responses, disturbing antibody recognition by steric hindrance of the binding site.

Since the N-X-T motif at residue 158–160 started to increase in prevalence worldwide in 2015, the WHO still recommended the vaccine strain (A/Hong Kong/4801/2014) until September 2017 (Allen and Ross, 2018). In Guangdong, China, the N-X-T motif became dominant in 2016. Before September 2017, the potential glycosylation site at residue 158 resulted in disruption of antibody recognition. Despite the WHO updating the vaccine strain to A/Singapore/INFIMH-16-0019/2016 bearing a glycosylation site at residue 158, an H3N2 outbreak still occurred in Guangdong, China in 2019. This may affect the vaccine strategy of local government or represent new drift mutations in H3N2 viruses. However, the N-X-T motif at residues 158–160 and the resulting potential glycosylation site will be a focus in Guangdong, China in the future.

A limitation of our study was that we lacked a large number of clinical specimens to be analyzed. In addition, we will continue to test protective antibody responses against the circulating H3N2 virus by generating mAbs and human serum.

## Conclusion

Glycosylation at HA1 residues 158–160 of circulating H3N2 viruses in Guangdong, China in 2017 blocked binding to RBC receptors. Introduction of glycosylation sites and glycoform variation can affect protective antibody responses, and thus are critical for guiding vaccine strain selection in specific areas.

## Data Availability Statement

The datasets generated for this study are available on request to the corresponding author.

## Ethics Statement

The studies involving human participants were reviewed and approved by Ethics Committee of The First Affiliated Hospital of Guangzhou Medical University. The patients/participants provided their written informed consent to participate in this study.

## Author Contributions

XW, WS, and J-RW contributed to conceive and design the experiments. ZZ and L-FY contributed to perform the experiments and wrote the original draft. ZL, XX, and ZY contributed to analyze the data and provide the data interpretation. All authors contributed to the study design, data interpretation and revisions to the text, and approved the final text and agreed to be accountable for the work.

## Conflict of Interest

The authors declare that the research was conducted in the absence of any commercial or financial relationships that could be construed as a potential conflict of interest.

## Footnotes

1 http://gisaid.org

## References

[B1] Abe Y. Takashita E. Sugawara K. Matsuzaki Y. Muraki Y. Hongo S. (2004). Effect of the addition of oligosaccharides on the biological activities and antigenicity of influenza A/H3N2 virus hemagglutinin. *J. Virol.* 78 9605–9611. 10.1128/jvi.78.18.9605-9611.2004 15331693 PMC514993

[B2] Allen J. D. Ross T. M. (2018). H3N2 influenza viruses in humans: viral mechanisms, evolution, and evaluation. *Hum. Vaccines* 14 1840–1847. 10.1007/s40266-018-0537-3 29641358 PMC6149781

[B3] Alymova I. V. York I. A. Air G. M. Cipollo J. F. Gulati S. Baranovich T. (2016). Glycosylation changes in the globular head of H3N2 influenza hemagglutinin modulate\\r receptor binding without affecting virus virulence. *Sci. Rep.* 6:36216. 10.1038/srep36216 27796371 PMC5086918

[B4] An Y. Parsons L. M. Jankowska E. Melnyk D. Joshi M. Cipollo J. F. (2019). N-Glycosylation of Seasonal influenza vaccine hemagglutinins: implication for potency testing and immune processing. *J. Virol.* 93:e01693-18. 10.1128/jvi.01693-18 30355697 PMC6321900

[B5] Beer K. Dai M. Howell S. Rijal P. Townsend A. R. Lin Y. (2018). Characterization of neutralizing epitopes in antigenic site B of recently circulating influenza A(H3N2) viruses. *J. Gen. Virol.* 99 1001–1011. 10.1099/jgv.0.001101 29944110 PMC6171714

[B6] Boeijen M. (2013). Glycosylation of the influenza A virus hemagglutinin protein. *Int. J. Nuclear Med. Biol.* 11 103–105.

[B7] Chambers B. S. Parkhouse K. Ross T. M. Alby K. Hensley S. E. (2015). Identification of Hemagglutinin Residues Responsible for H3N2 Antigenic Drift during the 2014-2015 influenza season. *Cell Rep.* 12 1–6. 10.1016/j.celrep.2015.06.005 26119736 PMC4487778

[B8] Cheng-Chi W. Juine-Ruey C. Yung-Chieh T. Che-Hsiung H. Yu-Fu H. Shih-Wei C. (2009). Glycans on influenza hemagglutinin affect receptor binding and immune response. *Proc. Natl. Acad. Sci. U.S.A.* 106 18137–18142. 10.1073/pnas.0909696106 19822741 PMC2775302

[B9] Cueno M. E. Shiotsu H. Nakano K. Sugiyama E. Kikuta M. Usui R. (2019). Structural significance of residues 158-160 in the H3N2 hemagglutnin globular head: a computational study with implications in viral evolution and infection. *J. Mol. Graph. Model.* 89 33–40. 10.1016/j.jmgm.2019.02.007 30849718

[B10] de Wit E. Munster V. J. van Riel D. Beyer W. E. Rimmelzwaan G. F. Kuiken T. (2010). Molecular determinants of adaptation of highly pathogenic avian influenza H7N7 viruses to efficient replication in the human host. *J. Virol.* 84 1597–1606. 10.1128/jvi.01783-09 19939933 PMC2812334

[B11] Deshpande K. L. Fried V. A. Ando M. Webster R. G. (1987). Glycosylation affects cleavage of an H5N2 influenza virus hemagglutinin and regulates virulence. *Proc. Natl. Acad. Sci. U.S.A.* 84 36–40. 10.1073/pnas.84.1.36 3467357 PMC304136

[B12] Gerhard W. Yewdell J. Frankel M. E. Webster R. (1981). Antigenic structure of influenza virus haemagglutinin defined by hybridoma antibodies. *Nature* 290 713–717. 10.1038/290713a0 6163993

[B13] Grimes S. E. (2002). “A Basic Laboratory Manual for the Small-Scale Production and Testing of I-2 Newcastle Disease Vaccine,” in *10. Haemagglutination test* (Rockville, MD: RAP publication). Available online at: http://www.fao.org/3/ac802e/ac802e00.htm#Contents

[B14] Harvala H. Frampton D. Grant P. Raffle J. Nastouli E. (2017). Emergence of a novel subclade of influenza A(H3N2) virus in London, December 2016 to January 2017. *Eur. Commun. Dis. Bull.* 22:30466. 10.2807/1560-7917.ES.2017.22.8.30466 28251889 PMC5356434

[B15] Hensley S. E. Das S. R. Bailey A. L. Schmidt L. M. Hickman H. D. Jayaraman A. (2009). Hemagglutinin receptor binding avidity drives influenza A virus antigenic drift. *Science* 326 734–736. 10.1126/science.1178258 19900932 PMC2784927

[B16] Hoffmann E. Neumann G. Kawaoka Y. Hobom G. Webster R. G. (2000). A DNA transfection system for generation of influenza A virus from eight plasmids. *Proc. Natl. Acad. Sci. U.S.A.* 97 6108–6113. 10.1073/pnas.100133697 10801978 PMC18566

[B17] Keil W. Geyer R. Dabrowski J. Dabrowski U. Niemann H. Stirm S. (1985). Carbohydrates of influenza virus. Structural elucidation of the individual glycans of the FPV hemagglutinin by two-dimensional 1H n.m.r. and methylation analysis. *EMBO J* 4 2711–2720. 10.1002/j.1460-2075.1985.tb03991.x 4054103 PMC554564

[B18] Knossow M. Daniels R. S. Douglas A. R. Skehel J. J. Wiley D. C. (1984). Three-dimensional structure of an antigenic mutant of the influenza virus haemagglutinin. *Nature* 311 678–680. 10.1038/311678a0 6207440

[B19] Lin Y. Wharton S. A. Whittaker L. Dai M. Ermetal B. Lo J. (2017). The characteristics and antigenic properties of recently emerged subclade 3C.3a and 3C.2a human influenza A(H3N2) viruses passaged in MDCK cells. *Influenza Other Respir. Viruses* 11 263–274. 10.1111/irv.12447 28164446 PMC5410720

[B20] Mair C. M. Ludwig K. Herrmann A. Sieben C. (2014). Receptor binding and pH stability - how influenza A virus hemagglutinin affects host-specific virus infection. *Biochim. Biophys. Acta* 1838 1153–1168. 10.1016/j.bbamem.2013.10.004 24161712

[B21] Melidou A. Gioula G. Exindari M. Ioannou E. Gkolfinopoulou K. Georgakopoulou T. (2017). Influenza A(H3N2) genetic variants in vaccinated patients in northern Greece. *J. Clin. Virol.* 94 29–32. 10.1016/j.jcv.2017.07.003 28734139

[B22] Nair H. Brooks W. A. Katz M. Roca A. Berkley J. A. Madhi S. A. (2011). Global burden of respiratory infections due to seasonal influenza in young children: a systematic review and meta-analysis. *Lancet* 378 1917–1930. 10.1016/s0140-6736(11)61051-9 22078723

[B23] Park A. W. Daly J. M. Lewis N. S. Smith D. J. Wood J. L. Grenfell B. T. (2009). Quantifying the impact of immune escape on transmission dynamics of influenza. *Science* 326 726–728. 10.1126/science.1175980 19900931 PMC3800096

[B24] Popova L. Smith K. West A. H. Wilson P. C. James J. A. Thompson L. F. (2012). Immunodominance of antigenic site B over site A of hemagglutinin of recent H3N2 influenza viruses. *PLoS One* 7:e41895. 10.1371/journal.pone.0041895 22848649 PMC3405050

[B25] Poucke S. V. Doedt J. Baumann J. Qiu Y. Matrosovich M. (2015). Role of Substitutions in the Hemagglutinin in the Emergence of the 1968 Pandemic influenza virus. *J. Virol.* 89 12211–12216. 10.1128/JVI.01292-15 26378170 PMC4645308

[B26] Schoenbaum S. C. (2004). The great influenza: the epic story of the deadliest plague in history. *J. Public Health Policy* 25 435–443. 10.1080/01947648.2015.1262197 28256945

[B27] Shinya K. Ebina M. Yamada S. Ono M. Kasai N. Kawaoka Y. (2006). Avian flu: influenza virus receptors in the human airway. *Nature* 440 435–436. 10.1038/440435a 16554799

[B28] Skehel J. J. Stevens D. J. Daniels R. S. Douglas A. R. Knossow M. Wilson I. A. (1984). A carbohydrate side chain on hemagglutinins of Hong Kong influenza viruses inhibits recognition by a monoclonal antibody. *Proc. Natl. Acad. Sci. U.S.A.* 81 1779–1783. 10.1073/pnas.81.6.1779 6584912 PMC345004

[B29] Skehel J. J. Wiley D. C. (2000). Receptor binding and membrane fusion in virus entry: the influenza hemagglutinin. *Annu. Rev. Biochem.* 69 531–569. 10.1146/annurev.biochem.69.1.531 10966468

[B30] Skowronski D. M. Sabaiduc S. Chambers C. Eshaghi A. Li Y. (2016). Mutations acquired during cell culture isolation may affect antigenic characterisation of influenza A(H3N2) clade 3C.2a viruses. *Eur. Commun. Dis. Bull.* 21:30112. 10.2807/1560-7917.ES.2016.21.3.30112 26836031

[B31] Tate M. D. Job E. R. Deng Y. M. Gunalan V. Maurer-Stroh S. Reading P. C. (2014). Playing hide and seek: how glycosylation of the influenza virus hemagglutinin can modulate the immune response to infection. *Viruses* 6 1294–1316. 10.3390/v6031294 24638204 PMC3970151

[B32] Wang Y. Zeng Z. Chen Q. Yan W. Chen Y. Xia X. (2019). Pterodontic acid isolated from Laggera pterodonta suppressed RIG-I/NF-KB/STAT1/Type I interferon and programmed death-ligand 1/2 activation induced by influenza A virus in vitro. *Inflammopharmacology* 27 1255–1263. 10.1007/s10787-019-00571-y 30783895

[B33] Wanzeck K. Boyd K. L. McCullers J. A. (2011). Glycan shielding of the influenza virus hemagglutinin contributes to immunopathology in mice. *Am. J. Respir. Crit. Care Med.* 183 767–773. 10.1164/rccm.201007-1184OC 20935106 PMC3159075

[B34] Westgeest K. B. Russell C. A. Lin X. Spronken M. I. Bestebroer T. M. Bahl J. (2014). Genomewide analysis of reassortment and evolution of human influenza A(H3N2) viruses circulating between 1968 and 2011. *J. Virol.* 88 2844–2857. 10.1128/jvi.02163-13 24371052 PMC3958060

[B35] Whittle J. R. Zhang R. Khurana S. King L. R. Manischewitz J. Golding H. (2011). Broadly neutralizing human antibody that recognizes the receptor-binding pocket of influenza virus hemagglutinin. *Proc. Natl. Acad. Sci. U.S.A.* 108 14216–14221. 10.1073/pnas.1111497108 21825125 PMC3161572

[B36] Yang H. Carney P. J. Chang J. C. Guo Z. Villanueva J. M. Stevens J. (2015). Structure and receptor binding preferences of recombinant human A(H3N2) virus hemagglutinins. *Virology* 477 18–31. 10.1016/j.virol.2014.12.024 25617824 PMC5696785

[B37] Zost S. J. Parkhouse K. Gumina M. E. Kim K. Diaz Perez S. Wilson P. C. (2017). Contemporary H3N2 influenza viruses have a glycosylation site that alters binding of antibodies elicited by egg-adapted vaccine strains. *Proc. Natl. Acad. Sci. U.S.A.* 114 12578–12583. 10.1073/pnas.1712377114 29109276 PMC5703309

