# Supplementary material for: Characterization and Evolutionary Analysis of a Novel H3N2 Influenza A Virus Glycosylation Motif in Southern China
Source: Front Microbiol. 2020 Jun 16;11:1318. doi: 10.3389/fmicb.2020.01318 (PMC7309185; doi:10.3389/fmicb.2020.01318)
Supplement: Supplementary file 1 [file Data_Sheet_1.PDF]

Supplementary Material

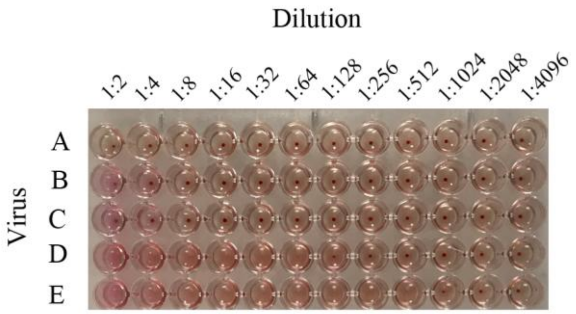

Figure S1 Hemagglutination titers of of H3N2 isolates

A: PBS; B: LZP; C:GMU-03; D: B10; E: HK4801

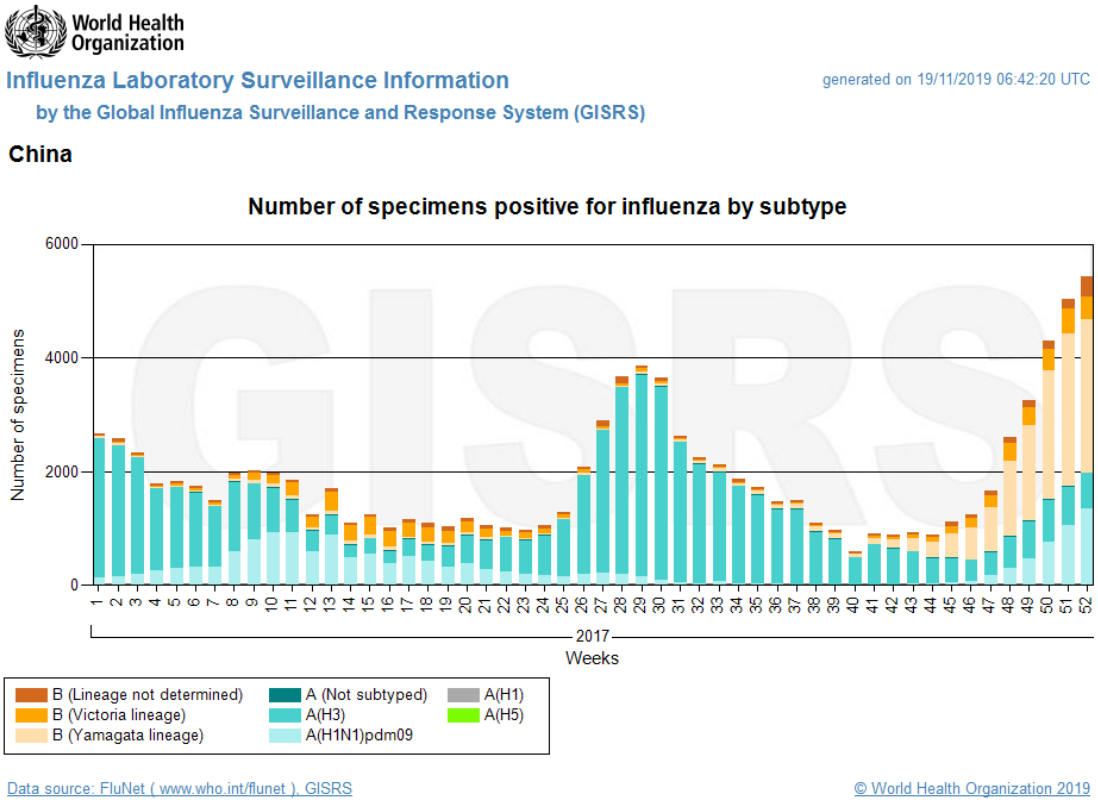

Figure S2 Number of specimens positive for influenza by subtype in China
